# Assessment of household preferences for net textile type (polyester versus polyethylene) for decision-making of the National Malaria Control Programme in Burkina Faso: methods for a quasi-experimental study

**DOI:** 10.1186/s12936-024-05105-8

**Published:** 2024-09-12

**Authors:** Aristide S. Hien, Hervé Hien, Herman Badolo, Serge M. A. Somda, Herman Bazié, Fidèle Isso Bacyè, Sidzabda Kompaoré, Matilibou Guira, Nicolas Meda

**Affiliations:** 1 Institut de Recherches en Sciences de la Santé, Centre National de la Recherche Scientifique et Technologique, Ouagadougou, Burkina Faso; 2 Institut National de Santé Publique, Ouagadougou, Burkina Faso; 3 Université Nazi Boni, Bobo-Dioulasso, Burkina Faso; 4 Centre Universitaire de Tenkodogo, Tenkodogo, Burkina Faso; 5 Programme National de Lutte Contre Le Paludisme, Ministère de la Santé et de l’Hygiène Publique, Ouagadougou, Burkina Faso; 6 Unité de Formation et de Recherche en Sciences de la Santé, Université Joseph Ki-Zerbo, Ouagadougou, Burkina Faso

**Keywords:** Insecticide-treated nets textile, Preference, Use, Mixed-model approach, National Malaria Control Programme, Burkina Faso

## Abstract

**Background:**

A quasi-experimental comparative trial will be designed in Burkina Faso. The study will compare the use and preferences for two groups types of insecticide-treated nets textile: polyester-based and polyethylene-based, according to their use and preferences in selected health districts. These health districts will be selected in three eco-climate zones (Sahelian, dry savannah and wet savannah) in the country. These findings will inform decisions on future net procurements for national malaria control programme in 2025.

**Methods:**

Quantitative surveys and qualitative data collection will be carried out to gather information on the type of net textile most commonly used and preferred by the community. They will be performed between the end of the dry season and the early rainy season. The quantitative surveys involved household interviews with households and individuals’ questionnaires, while the qualitative data collection involved in-depth individual interviews and focus group discussions to explore and clarify some key evaluation criteria. A total of 9450 insecticide-treated nets were surveyed for quantitative survey purposes. For the qualitative study, 48 in-depth individual interviews and 12 focus group discussions were carried out. A mixed model approach combining the results from quantitative surveys and qualitative studies will be used for decision-making on the type of insecticide-treated net preference.

**Conclusion:**

This methodological approach will be used by the National Malaria Control Programme to conduct this study on determinants of net use in Burkina Faso in order to provide robust evidence across diverse settings. This mixed-methods approach for data collection and analysis could be used in other countries to provide evidence that would help to increase the uptake of insecticide-treated nets, the main vector control tool in Africa.

## Background

Burkina Faso, a landlocked country in Central West Africa, faces a major public health issue related to endemic malaria [1]. The 2017–2018 Malaria Indicator Survey (MIS) revealed that the incidence of malaria among children aged between 6 and 59 months ranged from 7% in the central region to 39% in the southwestern region, as measured by microscopy [1, 2]. Malaria is seasonal in Burkina Faso, with the peak occurring between June and October. The duration of the rainy season varies across the country, impacting the incidence of seasonal malaria transmission in different geographical areas. In the north, the rainy season persists for up to 3 months, while in the central region, it endures for 6 months; in the south, it does so for 9 months.

The country deploys insecticide-treated nets (ITNs) as the primary method for preventing malaria. The National Malaria Strategic Plan for 2021 to 2025 outlines three approaches to ensure that ITNs are accessible to the entire population: (1) the free distribution of ITNs through national campaigns, (2) the free distribution of ITNs as part of routine antenatal care and expanded immunization programmes in all public facilities, and (3) the sale of ITNs by the private sector [3]. In 2010, the National Malaria Control Programme (NMCP) and its partners carried out the initial mass distribution campaign of insecticide-treated nets throughout the country [4]. Three additional campaigns were conducted in 2013, 2016, and 2019 [3]. The proportion of households with at least one ITN for every two individuals rose from 19% in the 2010 DHS to 41% in the 2021 DHS and population access to ITN increased from 36 to 64%. The percentage of the population who slept under ITNs also significantly increased from 31 to 61% in the same period. The percentage of ITNs used the previous night ranges from 75 to 96% in DHS and MIS data, and in the 2021 DHS, ITN use: access ratio was 0.95, indicating a high rate of people using nets among those with access (itnuse.org). These ITN use indicators demonstrate improvement, but overall ITN use remains below expectations as outlined in the National Malaria Control Strategic Plan 2016–2020 [5]. The plan targets a minimum of 90% of the population—100% for children less than 5 years of age—and 100% for pregnant women in Burkina Faso to sleep under ITNs [3].

The MIS and DHS conducted in Burkina Faso also provide data on the use rates of ITN for both polyester and polyethylene textiles. The textile can be determined from the brand of the ITN recorded in the survey. Overall, 85%, 96%, 81%, and 91% of the polyester nets were used the previous night in the DHS 2010, MIS 2014 and 2018, and DHS 2021 surveys, respectively, 82%, 97%, 72%, and 91% of polyethylene nets were used. However, to explore ITN use trends more robustly, additional covariates are needed to understand the potential differential usage of ITNs from different textiles, especially given that the context of Burkina Faso, which includes socioeconomic, health, environmental, and demographic factors, has undergone significant changes since 2015. These changes include major population movements, increases in the daytime and nighttime temperatures, and new economic practices that could impact the use of two types of net textiles. The lack of use of certain insecticide-treated nets (ITNs) may be associated with extrinsic factors (related to the mosquito net) and intrinsic factors (linked to community perceptions). One possible extrinsic factor may be attributed to the mesh material of the mosquito net. The choice of fabric, either polyester or polyethylene, significantly affects its usage and acceptance within communities, based on its texture [6, 7], visual appeal, or mesh size [6–8]. There is anecdotal and qualitative evidence suggesting that some households might favor softer polyester insecticide-treated nets (ITNs) over polyethylene ITNs, which can give rise to a "stiffer" sensation [6, 7]. Several factors may affect the acceptability of bed nets, including their shape (rectangular or conical) [9–12], color (white, green, or blue) [13–15], and community perceptions. Factors related to community perceptions are essentially a lack of awareness or key information that needs to be communicated to communities. However, the lack of awareness or information among communities can also contribute to the low usage of these nets. In fact, various studies have linked the limited usage of these nets to the practical and technical challenges associated with securing the net above the mat, the house's design [16, 17], the sensation of suffocation and discomfort resulting from high temperatures, even at night, and the preference for using local, herbal repellents [17, 18] against mosquitoes. Socioeconomic factors, such as high household economic status and access to healthcare and education, have been proven to be crucial in predicting ITN ownership and usage. However, the importance of purchasing power diminishes when nets are distributed for free or with heavy subsidies [19, 20]. Ethnicity can also play a significant role, with individuals from pastoral or seminomadic lifestyles being less likely to own or use an ITN than individuals from sedentary farming communities [21–23]. Studies also indicate that prioritizing the use of insecticide-treated nets (ITNs) for children under five years of age and women is common practice, with older children and adolescents being deprioritized, particularly when there is a shortage of ITNs and when sleeping spaces are shared [22].

Global procurement policies require that NMCP requests for ITNs of a single textile be justified by robust evidence that shows that there would be a significant reduction in ITN usage if the less-preferred textile was procured. However, previous studies carried out in Burkina Faso on the use of ITNs and preferences for a specific textile have all been cross-sectional studies based on data from large national household surveys [23–25]. These studies do not take into account people's perceptions and experiences from a qualitative approach. It is therefore important to complement the quantitative data collected with qualitative data via a combined approach to decision-making. In addition, no prospective studies have been carried out in Burkina Faso on the way that ITN preferences could influence the durability of nets under real conditions. To assess this phenomenon, the NMCP planned an evaluation to explore the acceptability and use of polyester and polyethylene ITNs in the community, with ITNs distributed during the 2022 mass distribution campaign. The overall aim was to gather empirical data on ITN textile preferences by communities that would ultimately improve accessibility to healthcare products by providing effective ITNs to the population. The secondary objectives were i) to assess the ideational factors, practices and perceived effectiveness of insecticide-treated bed nets (ITNs) for various textiles in Burkina Faso through quantitative data analysis and ii) to evaluate individual perceptions and preferences regarding ITN use and ownership in Burkina Faso through qualitative study data analysis. The results will inform decisions for future ITN procurements for the NMCP and for the population in 2025.

## Methods

### Study design

This study will be a comparative quasi-experimental cross-sectional design. Dual-active ingredient ITNs (chlorfenapyr-pyrethroid) and PBO-pyrethroid ITNs were distributed during universal coverage campaigns conducted in 2022, with four brands fabricated with two different textiles (polyester or polyethylene). Distribution was based on malaria transmission intensity and insecticide-resistance profiles at the district level. A quasi-experimental design was used because randomization by net type was not feasible in the context of the 2022 mass distribution.

**Fig. 1:**
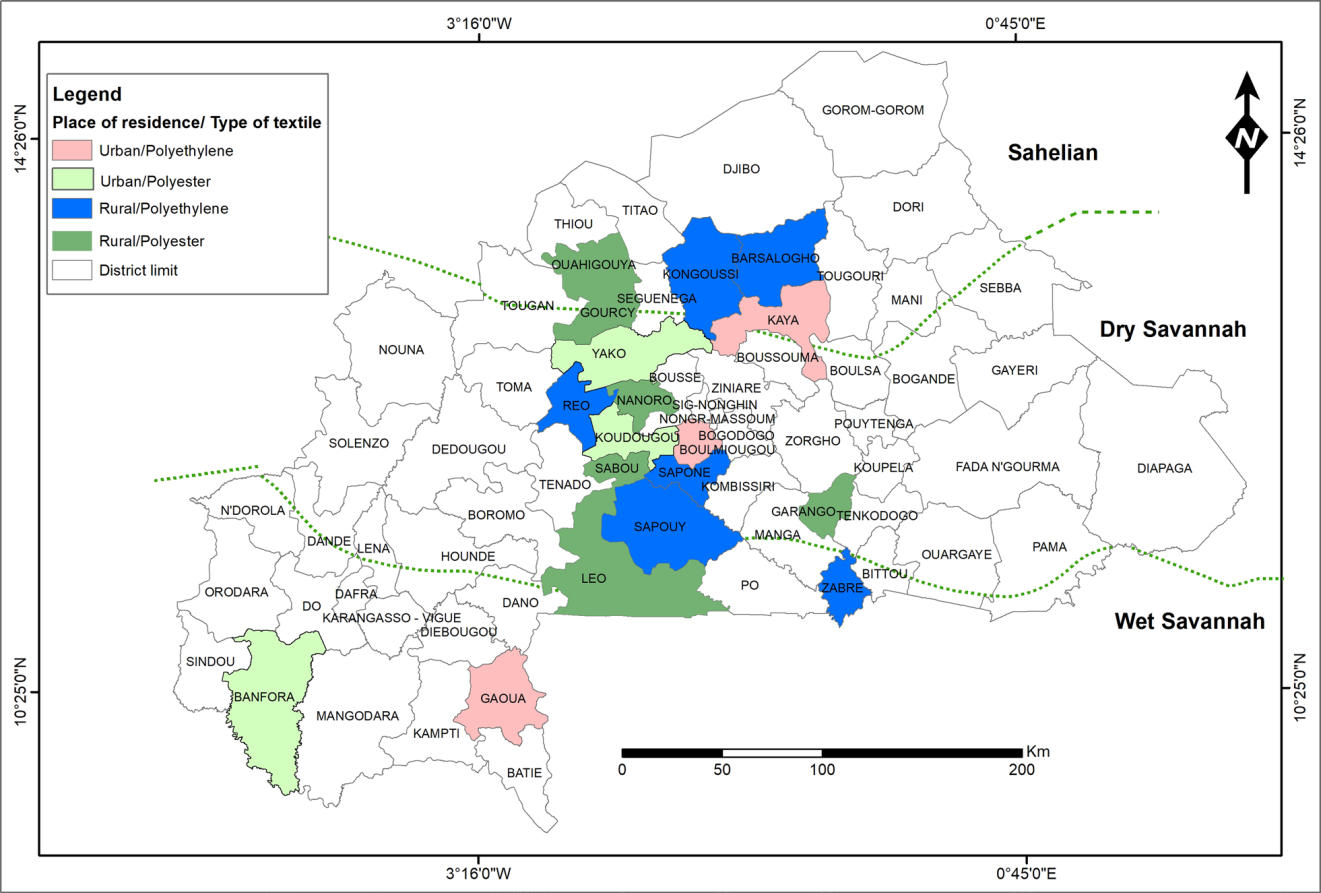
Map showing the selection method of study sites in climate areas

Eighteen study districts were selected, six per eco-climate (sahelian, dry savannah and wet savannah zones). In each eco-climate zone, the six districts will be further stratified by type of residence (two urban and four rural) (Fig. 1) to account for different seasonal patterns of ITN use and socio-geographic factors. Health districts with similar characteristics, but which had received different types of ITNs will be identified within each setting. Districts where polyethylene ITNs were distributed will be paired with a neighbouring district where polyester ITNs were distributed. Within each district, fourteen (14) enumeration zones (clusters or EZ) will be selected using proportional probability sampling, using the 2019 General Census of Population and Housing (GCPH-2019) conducted by the National Institute of Statistics and Demography (NISD). Within each EZ, fifteen (15) households will be selected using simple random sampling after a census of all households in the EZ. Data collection will be carried out using computer-assisted personal interviewing (CAPI) methods. This method has the advantages of helping interviews become more dynamic by reducing the risks associated with lost or damaged paper forms and minimizing data entry errors. However, during the field phase, the data collected will be regularly sent to the server, enabling errors to be detected and the returns to fields to be organized.

For a better understanding of the underlying reasons for net preferences, the systematic collection of quantitative data will be combined with qualitative data with key stakeholders identified in the study areas. In this way, all the questions will be systematically addressed using both quantitative surveys and qualitative research approaches with the ITN as the unit observation.

## Study population and sample size

### Quantitative surveys

The target population was all households residing in the study areas in Burkina Faso. Eligible respondents will be men or women aged at least 18 years, residing in study sites since the ITN distribution. Respondents provided informed consent to participate. In each selected household, the head of household or his or her representative will be eligible to respond to the household questionnaire, and two randomly selected members from this the household (man or woman) will be surveyed. The head of household or his or her representative will be interviewed using the household questionnaire, while the individual questionnaire will be administered to a selected man or woman different from those already selected for the household surveys.

Furthermore, the sample size of the ITNs that must be surveyed will be determined by conducting a likelihood ratio test to compare the proportions of two samples, one from districts using polyethylene and the second from districts using polyester. The first and second type errors will be set at 5% and 20%, respectively, to achieve a satisfactory balance between precision and logistical feasibility of the survey. The ratio of the indicator between both indicators (ITN use and preference) will be considered to be 1.09. This will allow to the selection of 583 households by group (polyester and polyethylene) and a total of 1,166 households by eco-climate area. This sample size will be increased to 1,260 per eco-climate area considering a nonresponse rate of 5%. After sample size correction, a total of 3,780 households will be randomly selected in each eco-climate area. Otherwise, based on the mean number of people per household, which is 5.2, and one ITN for two people in each household, as indicated by GCPH 2019, approximately 2.5 nets will be required per household in this study. Therefore, a total of 9450 ITNs will be surveyed for study purposes (Table 1).

**Table 1:** Sampling of insecticides-treated nets by zone and at national level

Eco-climate areas	Type of ITNs textile	Place of residence		Total ITNs	Total Village	Total Household	Mean anticipated ITN per Household	Target number of ITNs to survey
		Urban	Rural					
Sahelian	Polyethylene	1	2	3	42	630	2.5	1260
	Polyester	1	2	3	42	630	2.5	1260
Dry savannah	Polyéthylene	1	2	3	42	630	2.5	1260
	Polyester	1	2	3	42	630	2.5	1260
Wet savannah	Polyethylene	1	2	3	42	630	2.5	1260
	Polyester	1	2	3	42	630	2.5	1260
Nation level	Polyethylene	3	6	9	126	1890	2.5	4725
	Polyester	3	6	9	126	1890	2.5	4725
Total		6	12	18	252	3780	2.5	9450

### Qualitative data

The collection of qualitative data will help to confirm or better understand the quantitative data. In fact, in the given responses of interviewees from the quantitative data collection, some answers need further clarifications, and those clarifications could be obtained through the focus group discussions with men and women or through in-depth individual interviews with people who have a great deal of experience in community health, such as community health workers. The qualitative data collection will involve community-based health workers (CHWs) and male or female heads of household from the study areas. Indeed, heads of household are generally the primary decision-makers in terms of net use, or people responsible for ensuring that nets are used correctly in the community. In this study, two qualitative data collection approaches will be implemented: focus group discussions and in-depth individual interviews (IDIs). It will be challenging to understand people’s perceptions of the IDIs who justify the option of interviewing them to complete and better explain the social occurrence of certain quantitative variables. The objective will be, therefore, to collect all the common information discussed in households about mosquito nets that the focus group could not reveal. The interviews with the CHWs will fulfill the function of triangulating information. The CHWs thus will constitute a control and verification group for data collected from the households during focus group discussions. Furthermore, the focus group discussions will be mixed and include both men and women who are heads of household. The eligibility criteria for the selection of focus group participants will include residing in a district or EZ to be surveyed and not participating in the quantitative survey. The eligibility criteria for the in-depth individual interviews will include residing in the selected EZ, length of service (from oldest to youngest) and sex. The criteria for the in-depth individual interviews will be based on belonging to the selected EZ, the length of service (from oldest to youngest) and sex. The CHWs will be the target audience for answering the questions.

In-depth interviews (IDIs) will be conducted with four CHWs in each urban district per eco-climate zone (two per textile) and two CHWs from the rural districts in each eco-climate zone. Thus, four CHWs will be interviewed per textile per eco-climate zone, for a total of 24 CHW. One focus group discussion (FGD) will be conducted in all urban districts and in half of the rural district, consisting of 12 people (men and women mixed) per session. In total, 12 FGDs will be conducted (four per eco-climate zone), for a total of 144 participants (Table 2).

**Table 2:** Summary of qualitative data sampling

In-depth individual interviews						
Eco-climate areas	Type of ITNs textile	Place of residence		Total CHWs/ITN	Total Heath districts/ITN	Total CHWs interviewed
		Urban	Rural			
Sahelian	Polyethylene	2	2	4	2	8
	Polyester	2	2	4	2	8
Dry savannah	Polyéthylene	2	2	4	2	8
	Polyester	2	2	4	2	8
Wet savannah	Polyethylene	2	2	4	2	8
	Polyester	2	2	4	2	8
Nation level	Polyethylene	6	6	12	6	24
	Polyester	6	6	12	6	24
Total		12	12	24	12	48

Focus group discussions						
Eco-climate areas	Type of ITNs textile	Place of residence		Number FGD / ITNs sites	Number of participants/ FGD	Total participants
		Urban	Rural			
Sahelian	Polyethylene	1	1	2	12	24
	Polyester	1	1	2	12	24
Dry savannah	Polyéthylene	1	1	2	12	24
	Polyester	1	1	2	12	24
Wet savannah	Polyethylene	1	1	2	12	24
	Polyester	1	1	2	12	24
Nation level	Polyethylene	3	3	6	36	72
	Polyester	3	3	6	36	72
Total		6	6	12	72	144

CHWs: Communty health workers; FGD: Focus group discussion

## Data collection

### Quantitative surveys

Quantitative data collection will start by delimiting the EZ and listing the households in the EZ before systematically selecting fifteen (15) households from among those listed. In addition, in each household selected, the agents will administer the questionnaire to the head of household or his or her representative. Basic demographic information will be collected from the head of the household or from adults older than 18 years. However, households for which the interviewers are unable to find the head or his or her representative and households for which the head or his or her representative did not give their consent will be replaced after notification.

Two types of structured questionnaires will be used to collect data: the household questionnaire and the individual questionnaire. The individual questionnaire will be administered to either a woman or a man from the household already selected who was at least 18 years old. The content of these questionnaires will be based on the standard Malaria Behavior Survey (MBS) questionnaires (https://malariabehaviorsurvey.org/) [26]. For the purposes of this survey, a household is defined as a group of people living under the same roof and sharing meals. The household questionnaire will be administered to the head of household or another adult member of the household (his or her representative) to survey the use and preference of ITNs, which are key outcomes of this study. In addition, other ranges of factors related to ITN management and usage will be discussed, including possession habits, household member usage, ITN management, usage-to-access ratio, and usage patterns among survey participants. The household survey will be used to record all household members and visitors who had spent the night before the interview and will also be used to identify women and men eligible for individual interviews. The household surveys will aim to provide insight into household living conditions, including the housing characteristics, assets, household composition and sociodemographic characteristics of the respondents. In fact, the use and preference of ITNs are often associated with the factors cited above. The questionnaire also collected attitudes toward the use of ITNs using a six-item Likert scale with three possible responses, excluding the neutral option − 1 to + 1, with those having more positive attitudes becoming significantly more positive (score ≥ 1.0) [25]. Otherwise, the individual man/woman questionnaire will be used to collect information on knowledge, attitudes, perceptions and practices related to malaria, the identification of awareness channels and the use of ITNs. Beyond the demographic data collected, individual and household questionnaires will serve to collect data on ITNs, such as the number of ITNs received during the campaign, the reasons for not receiving them or not participating in the campaign, the perceived effectiveness of ITNs by communities, and the reasons for the loss of ITNs received during the campaign.

### Qualitative data collection

Two types of guides will be used to collect qualitative data: one for in-depth individual interviews and one for focus groups. The data will be collected through focus group discussions with men and women and in-depth individual interviews with CHWs. The qualitative data will be collected during the same period immediately after the quantitative data collection. The sex distribution of the CHWs recruited will be alternated according to place of residence, such that when the first CHW will be recruited as a woman in an urban area, the second recruitment will be made in a rural area, during which a man in the same eco-climate area will be selected. In-depth interviews with CHWs will be focused on CHW experiences with ITNs, their observations on ITN use and CHWs will also assist in identifying FGD participants.

CHWs also will assist in identifying FGD participants. The focus group discussion questions will assess attitudes toward ITNs and the effectiveness of using or not using ITNs to protect against malaria, the preference for a specific type of ITN textile, and the reasons for any preferences. The supervisor of the research team will lead the FGDs in collaboration with assistant interviewers who will translate them into other languages spoken at the study sites. All interviews of groups will be recorded.

## Data management

The collection of quantitative data will be carried out using computer-assisted personal interviewing (CAPI) methods. This has the advantage of making the interviews more dynamic and reducing the risks associated with loss or deterioration of paper forms. The use of data sets will also reduce data entry errors by investigators. An initial pretest will be then conducted to confirm whether the data transfer is correct and whether the server works very well. The collected data will be regularly sent to the server during the field phase. This approach will allow us to detect errors and manage feedback from the field. The data will be processed by checking for missing data, outliers, illegal values, etc. The process also will include the creation of indicators through calculations or data aggregation. This processing will allow the production of final data sets for analysis. Qualitative data will be collected through the facilitation of focus groups with men and women and in-depth interviews with CHWs. Only the data managers will have access to the data on the server.

## Data analysis

### Quantitative surveys

The data collected will be organized in a common database. Data processing will involve checking for missing data, outliers, illegal values, etc., and will involve compiling indicators by means of calculations or data aggregation. Univariate analysis will be used to determine the proportion of respondents who self-reported using each type of ITN textile the night before the survey and the proportion of respondents who preferred polyester or polyethylene or who won’t have any preference. The multivariate analysis will consider textile, gender, eco-climate area, region, place of residence (urban or rural), education and socioeconomic status. Data processing will enable the creation of finalized data sets for analysis. The quantitative data will be compared using STATA version 10.1.

### Qualitative data collection

The qualitative data will be transcribed as they were collected to enable the coordination team to validate the data and proceed according to the interview-transcription-validation strategy. The data will be initially organized by eco-climate zone. The interviews will be then coded and encoded. Following this process, the themes according to the research objectives and the themes emerging from the interviews will be identified. The themes will be, then, isolated by corpus according to eco-climate zone, followed by a comparison of themes and content according to the eco-climate zones of interest. The analysis of the data will be thematic, with the aim of explaining and complementing the quantitative aspects.

The qualitative data will be analysed using the NVivo 2020 software package.

A mixed model approach combining results from quantitative surveys and qualitative studies will be used for decision-making. The method described is completely new. The data will be analysed according to the standard plan described below:

#### Primary endpoint criteria

The decision criteria for the primary endpoint will be whether the type of ITN textile has a positive effect on the overall ITNs use in term of significant proportion of one type of ITNs textile self-reported used the night before the survey. For that purpose, the quantitative study shall determine the statistical frequency of each type of ITN textile self-reported used the night before the investigators' attendance in univariate and also multivariate logistic regression analysis.

#### Analysis of primary endpoint

The primary outcome will be determined for the whole population and specifically for ITNs. A logistic regression model will be used to compare ITNs. This model will be adjusted for potential factors confounders. The main factors confounders will be (1) sex (male/female); (2) socioeconomic status (education level, religion); (3) residence (urban/rural); (4) ecoclimatic or epidemiological zone (Sahel, dry savannah and wet savannah area); and (5) wealth quintile.

#### Decision-making point

Positive responses to a single type of ITN textile from quantitative interviews will be used to determine which ITN textile will be most used and preferred by respondents. The qualitative data surveys would support the decision-making process by analysing the trends of the responses from the FGD or IDIs for the same question. However, in the case of discordant results, additional analyses including attitudes toward care and repair of a comparable net type will be performed to support decision-making in such a situation.

## Discussion

This study aims to identify the most appropriate ITN textile for the population of Burkina Faso who receives ITNs through mass distribution campaigns. Burkina Faso underwent four universal ITN distribution campaigns from 2010 to 2019 [4]; however, the country has not been able to meet the expected threshold set by the World Health Organization for ITN usage, which is at least 80%. These hypotheses are linked to the gap observed during distribution campaigns, but recent studies carried out in certain regions (Côte d'Ivoire, Cameroon) tend to underpin ideational factors linked to the use of ITNs by communities [26]. To maximize ITN use in Burkina Faso, this study aimed to determine the intrinsic factors linked to mosquito nets (net textiles), which could be one of the probable causes of nonuse of mosquito nets. In fact, the type of mosquito net fabric, polyester or polyethylene, plays a predominant role in its use and acceptability by communities, whether it is to the touch [5] or the size of the mesh [7]. Indeed, anecdotal reports and qualitative observations have indicated that in some locations, households may prefer softer polyester ITNs to polyethylene ITNs, which can have a “harder” feeling [5]. With the support of technical partners such as the Global Fund, the National Malaria Control Programme initiated this study in conjunction with the distribution campaign to be carried out in 2022 to provide scientific evidence for the choice of a type of mosquito net fabric that will be most accepted by the population. The aim of this study was to provide scientific evidence for determining the most accepted type of mosquito net fabric used by the population. It will be imperative to conduct this study and identify any intrinsic factors related to mosquito nets (in textile material) that could cause non usage.

The quasi-experimental model uses quantitative and qualitative data collection to address the issue of inadequate ITN ownership use, potentially linked to the net type. By definition [27], this approach does not involve randomization. Quasi-experimental studies assess the association between an intervention and an outcome using experiments in which the intervention is not randomly assigned [28]. Quasi-experimental studies can be classified into three broad categories: interrupted time series, studies with control groups and studies without control groups [29]. Quasi-experimental methods identify a control group that should be as close as possible to the experimental group in terms of initial (preintervention) characteristics. In addition, this type of study design is less expensive and has higher external validity [30]. Furthermore, quasi-experimental studies can use observations collected retrospectively or prospectively or a combination of the two. Due to the lack of research funding in Burkina Faso, retrospective data from the Malaria Indicator Survey (MIS) or the Demographic Health Survey are needed to provide scientific evidence on net coverage in Burkina Faso. Although the MIS and health indicator surveys provide some insight into the extent of ITN use in the country [23], they lack the thoroughness needed to confirm the actual state of ITN use. As several authors have noted, the data from this study were retrospective and cross-sectional, which means that they cannot be used for cause-and-effect analyses. In addition, the effectiveness of ITN may be underestimated because the data collection period may not coincide with the period of malaria transmission [24, 25].

Furthermore, these findings do not consider the influence of household dynamics on the use of bed nets among household members. As a result, population-based studies need to use a prospective methodology that reflects real-world scenarios, highlighting the importance of quasi-experimental methods. The results of this research could lead to the development of a national malaria control programme with scientific support for the selection of bed net textiles suitable for the context of Burkina Faso. This study aims to offer a rigorous and detailed methodology for other countries facing the challenge of low ITN use related to textile types of decision-making.

## Conclusion

The suboptimal use of insecticide-treated nets (ITNs) in households may be linked to the influence of user preferences on net textiles (polyester or polyethylene). Consequently, the National Malaria Control Programme will conduct a study to identify the determinants of ITN use in Burkina Faso to yield reliable evidence across diverse settings. Such an analytical approach could be applied in other countries to generate evidence that would contribute to increasing the use of nets, the most important vector control tool in Africa.

## Data Availability

No datasets were generated or analysed during the current study.

## Abbreviations

CAPI: Computer-Assisted Personal Interviewing
CHWs: Community health workers
FGD: Focus group discussion
GCPH: General census of population and housing
IDI: In-depth interview
INSD: Institut National de la Statistique et de la Démographie
ITNs: Insecticide-treated Nets
MIS: Malaria indicator surveys
NMCP: National Malaria Control Programme
ONSP: Observatoire National de Santé Publique
